# Detection of preperimetric glaucoma using Bruch membrane opening, neural canal and posterior pole asymmetry analysis of optical coherence tomography

**DOI:** 10.1038/srep21743

**Published:** 2016-02-17

**Authors:** Rui Hua, Rita Gangwani, Lei Guo, Sarah McGhee, Xiaoli Ma, Jun Li, Kai Yao

**Affiliations:** 1 Department of Ophthalmology, First Hospital of China Medical University, Shenyang, China; 2 Department of Ophthalmology. The University of Hong Kong, Hong Kong, China; 3 Ophthalmology and optometry center, First Hospital of China Medical University, Shenyang, China; 4 Department of Ophthalmology and Visual Science, Yale University School of Medicine, New Haven, CT 06511, USA

## Abstract

We analysed retinal nerve fibre layer (RNFL) defects in eyes with normal circumpapillary RNFL (cpRNFL) thickness using posterior pole asymmetry analysis (PPAA) and investigated the parameters of Bruch membrane opening (BMO) and neural canals using enhanced depth imaging spectral domain optical coherence tomography (EDI-SDOCT). A total of 112 preperimetric glaucomatous eyes of 92 patients were examined to obtain cpRNFL thickness using SD-OCT. Posterior pole asymmetry analysis (PPAA) and central cross-sectional images of the optic nerve head (ONH) were obtained using EDI-SDOCT. Minimal and horizontal distances between the BMO and ONH surfaces (BMOM, BMOH) and the terminal of retinal pigment epithelium (RPE) and ONH surfaces (RPEM, RPEH) were measured. The distribution of the absolute black cells in PPAA was more concentrated in eyes with “U”-shaped neural canals (p < 0.0001). The area under the receiver operating characteristic curve of the ratio of RPEM to RPEH (RPE-R, 0.771 ± 0.08) was significantly larger than the ratio of BMOM to BMOH (BMO-R, 0.719 ± 0.009) for PPAA results. A U-shaped neural canal, lower ratio of RPEM to RPEH, and lower ratio of BMOM to BMOH were considered early indicators of RNFL defects in preperimetric glaucomatous eyes with normal cpRNFL.

Glaucoma is an optic neuropathy with chronic neurodegenerative changes and progressive degeneration of the retinal ganglion cell (RGC) axons^1^, leading to the loss of visual field. The detection of early stage glaucoma, i.e., preperimetric glaucoma, has attracted increasing attention. However, the identification of preperimetric glaucoma via retinal nerve fibre layer defects (RNFLDs) remains controversial in clinical practice. Red-free reflectance imaging is used in the detection of RNFLD with high accuracy^2^, but the data cannot be analysed quantitatively, and the image quality is affected by many factors, such as cataract, leading to signal attenuation. Spectral domain optical coherence tomography (SD-OCT) is a relatively new imaging approach for the diagnosis and management of glaucoma^3^. Unfortunately, previous studies demonstrated that measurement of the circumpapillary retinal nerve fibre layer (cpRNFL) using SD-OCT provided unsatisfactory diagnostic capability and exhibited only moderate sensitivity in the detection of some cases with early RNFLD^4^,^5^ despite of its potential to recognize these defects. Normal cpRNFL was observed in many eyes with preperimetric glaucoma, supporting the development of new techniques and more reliable criteria for diagnosis.

The focus for the diagnosis of preperimetric glaucoma has recently moved from the peripheral retina to the macula. Glaucomatous damage involves a progressive loss of retina ganglion cells (RGCs). Therefore, observations of macular changes were added for structural assessments of glaucoma. RGCs are thickest in the perimacular area, and RGCs and RNFL constitute 30% to 35% of the retinal thickness in this region^6^. Therefore, damage in the RGCs is more readily detected in the macula than the peripheral retina. Early glaucomatous defects are generally localized to either side of the horizontal meridian, which allows the possible evaluation of asymmetry in hemifield macular thickness. This asymmetry has served as an early indicator of glaucomatous structural damage^7^. Recent studies found a significant reduction in the ratio of localized RNFL thickness to total retinal thickness during the early stages of glaucoma^8^, and the localized RNFL thickness exhibits a statistically significant structure-function association with the macular visual field (VF)^9^. Seo, *et al.*^10^ and Asrani *et al.*^11^ introduced a new method, termed as posterior pole asymmetry analysis (PPAA), using SD-OCT to test the difference between upper and lower macular thickness and detect localized RNFLD with higher sensitivity and specificity than the cpRNFL thickness^10^. They reported that the retinal thickness maps acquired through SD-OCT identified the presence of visible localized RNFLD and detected the thinning of RNFL, which is not otherwise detectable using stereo-photographic assessments^12^. However, these PPAA studies were generally conducted in abnormal cpRNFL eyes instead of normal cpRNFL eyes.

In addition to RNFL thickness, three anatomical structures of the optic nerve head (ONH) are of great concern in the discrimination of glaucomatous eyes, particularly preperimetric glaucoma, from healthy eyes. First, the neural canal opening (NCO), including the termination of the retinal pigment epithelium (RPE)/Bruch’s membrane (BM) complex, is not likely to change substantially with glaucoma progression^13^. Second, the BM opening (BMO), which is the internal entrance to the neural canal and an important structure of the ONH, is likely the true optic disc margin, and it was proposed as a stable zero reference plane for ONH quantification^14^,^15^. Third, anterior laminar displacement of lamina cribrosa (LC) tissue occurs after glaucoma surgery^16^. The LC is not a static structure, and it responds to intraocular pressure (IOP) changes. Once IOP rises in glaucomatous eyes, a cup excavation increase will be observed, primarily at the expense of prelaminar tissue thinning. LC thickness is significantly greater in normal subjects than glaucoma patients. The degree of thinning significantly correlates with glaucoma severity and increases with glaucomatous damage. Normal LC morphology in enhanced depth imaging SD-OCT (EDI-SDOCT) exhibits a “W” shape without an enlarged neural canal, but a “U” shape is observed when IOP increases, which indicates a thinner LC and enlarged neural canal. Therefore, LC plays a critical role in the pathogenesis of glaucoma, and EDI-SDOCT reveals the *in vivo* features of LC^17^. Agoumi *et al.*^18^ used manual delineation to evaluate the laminar and prelaminar surfaces and demonstrated changes in the LC and prelaminar tissue after IOP elevation.

However, the correlation between the morphology of ONH and PPAA has not been assessed in preperimetric glaucomatous eyes, particularly eyes with normal cpRNFL results. Therefore, we investigated subjects with preperimetric glaucomatous eyes and normal cpRNFL thickness. Eyes also exhibited typical wedge-shaped defects in red-free reflection and absolute black cells in PPAA. We further analysed PPAA patterns and the correlation between PPAA results and ONH morphology and also present new parameters for the diagnosis of glaucoma in patients with normal cpRNFL results.

## Results

### General information

**Figure 1:**
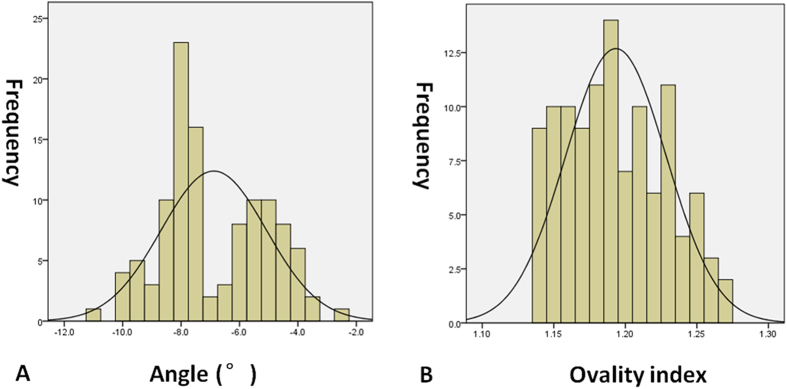
The distribution of ovality index (**A**) and the angle between the fovea and ONH centre relative to the horizontal axis (**B**).

This study included 112 eyes of 92 Chinese (northern Han race) patients with normal cpRNFL thickness from 28 males and 64 females with a mean age of 56.5 years (range: 19–77 years). The spherical equivalent (SE) was −0.75 diopters (range: −1.75– + 1.50 diopters) and the axial length was 23.73 mm (range: 22.58–24.35 mm). In addition, the angle between the fovea and ONH centre relative to the horizontal axis was −7.5°(range: −11.0°–2.4°), and the ovality index was 1.19 (range: 1.14–1.27) (Fig. 1). Moreover, the outlook of ONH was without torsion, and staphyloma. Besides, EDI-SDOCT identified that 60 of the 112 eyes exhibited “U”-shaped neural canals, and the remaining 52 eyes exhibited “W”-shaped neural canals (Table 1).

### The topography of PPAA

**Figure 2:**
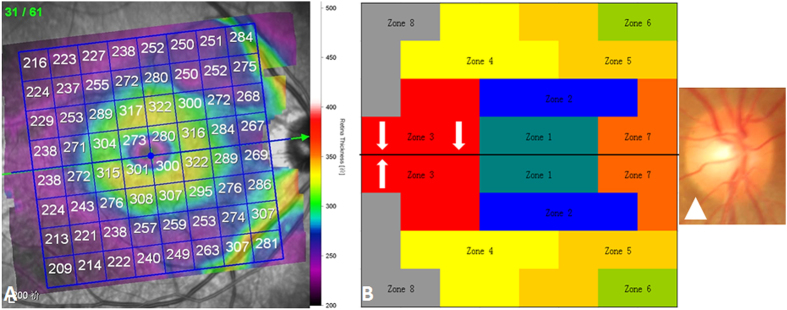
PPAA macular maps. (**A**) The posterior pole map provides a retinal thickness value of 64 (8 × 8) cells within each cell; (**B**) Eight zones in the macular thickness map, labelled with different colours, together with the fovea-disc axis (white arrow) and optic disc (white triangle).

**Figure 3:**
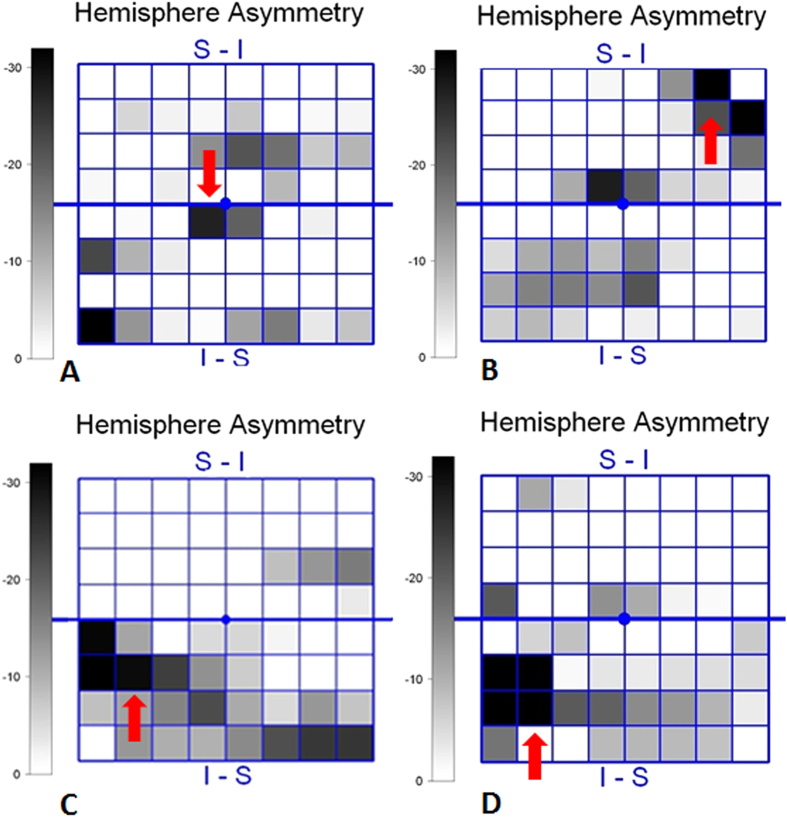
Consecutive absolute black cells in eyes in PPAA. (**A**) Single absolute black cells (red arrow); (**B**) Two consecutive absolute black cells (red arrow); (**C**) Three consecutive absolute black cells (red arrow); (**D**) Four consecutive absolute black cells (red arrow).

Three additional zones were added in each hemifield (Zones 6, 7, and 8) compared with previous studies of PPAA (Fig. 2). This addition may cover the entire retinal fibre region related to glaucoma injury. Zone 7 represents the papillomacular bundles. Zone 6 adds peripheral retinal nerve fibre information from the nasal and temporal sides of the retina. Zone 8 supplies peripheral retinal nerve fibre information from the temporal retina. The present study found that the absolute black cells were primarily concentrated in inferior Zones 4, 5, and 6 (45.8%) and superior Zones 5, 6, and 7 (38.9%). Zones 6, 7, and 8 contained 8.8%, 24.2%, and 2.2% black cells, respectively (Fig. 3). Zone 7 is very vulnerable to the glaucoma injury process.

**Figure 4:**
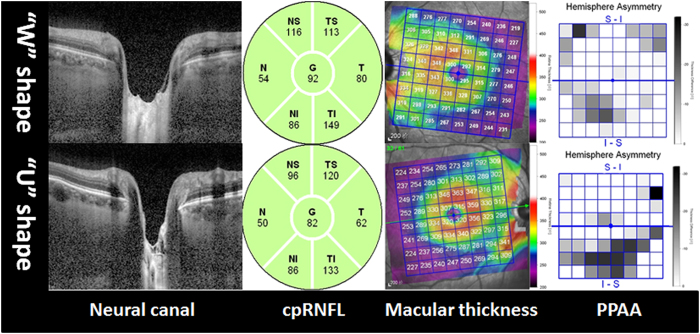
Comparison between “W”- and “U”-shaped neural canal eyes with normal cpRNFL (green colour). Absolute black cells in PPAA were obviously more concentrated in eyes with “U”-shaped neural canals than “W”-shaped neural canals. cpRNFL: circumpapillary retinal nerve fibre layer. The number in cpRNFL images revealed an average RNFL thickness in six directions (N: nasal, NS: nasal and superior, TS: temporal and superior, T: temporal, TI: temporal and inferior, NI: nasal and inferior) and the total average cpRNFL thickness (G). PPAA: posterior pole asymmetry analysis.

A total of 288 absolute black cells were counted in PPAA maps, and these cells concentrated in inferior Zones 4, 5, and 6 (45.8%) and superior Zones 5, 6, and 7 (38.9%) (Figs 2 and 3, respectively). PPAA (+) was identified in 64 eyes (36, 12 and 16 of these eyes contained 2, 3 and 4 consecutive absolute black cells, respectively). Fifty-two eyes exhibited “W”-shaped neural canals, and the other 60 eyes exhibited “U”-shaped neural canals (Fig. 4). Twenty, 8 and 4 eyes with “W”-shaped neural canals exhibited 1, 2 and 3 consecutive absolute black cells, respectively. The remaining 20 eyes did not contain any absolute black cells. Eight, 28, 8 and 16 eyes with “U”-shaped neural canal exhibited 1, 2, 3 and 4 absolute black cells, respectively. The absolute black cells in PPAA were obviously more concentrated in the eyes with “U”-shaped neural canals (r = 0.653, p < 0.0001). There was a significant positive correlation between the PPAA results and the morphology of the neural canal (“U” or “W” shaped) (r = 0.641, p < 0.0001).

**Figure 5:**
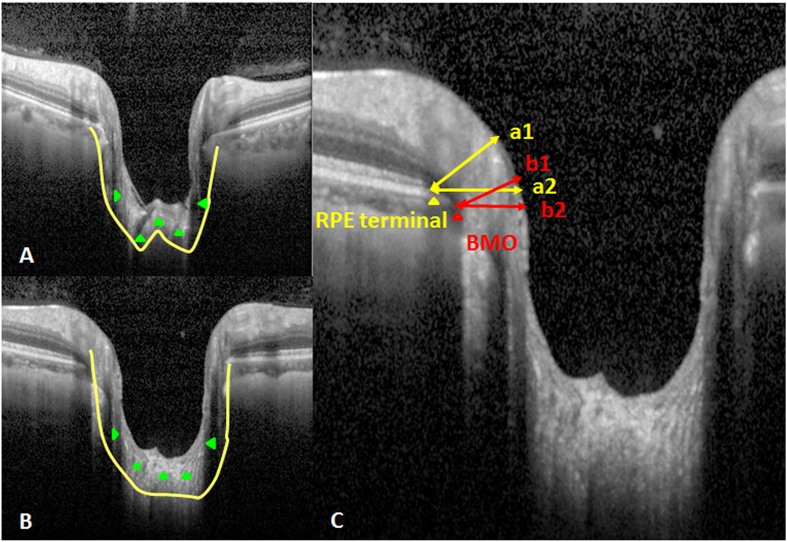
The morphology of ONH. (**A**) “W”-shaped neural canal (green triangle and yellow line); (**B**)“U”-shaped neural canal (green triangle and yellow line); (**C**) Measurement of NCO included RPEM (a1), RPEH (a2), BMOM (b1) and BMOH (b2); BMO (red triangle); terminal of RPE (yellow triangle).

**Figure 6:**
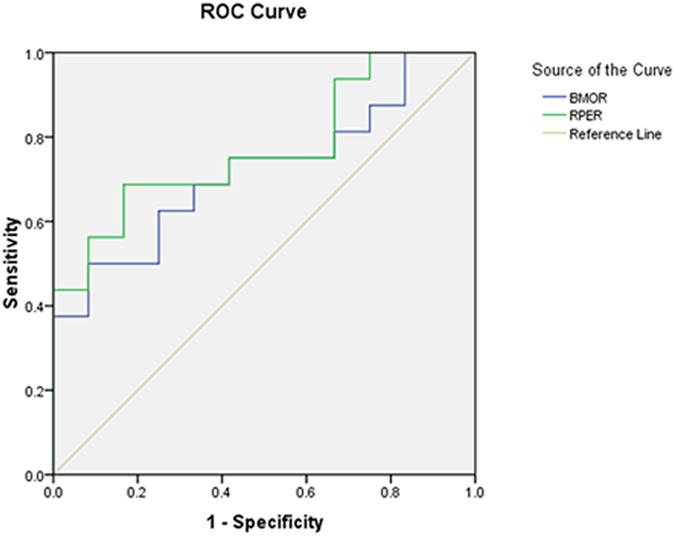
ROC curves of RPE-R and BMO-R. The AUROC of RPE-R was 0.771 ± 0.08, and the AUROC of BMO-R was 0.719 ± 0.009.

We measured the minimal and horizontal distances between the terminal of the RPE and ONH surfaces (RPEM and RPEH, respectively) and the BMO and ONH surfaces (BMOM and BMOH, respectively) to more quantitatively assess the NCO in these subjects (Fig. 5). The ratios of BMOM to BMOH (BMO-R) and RPEM to RPEH (RPE-R) were calculated. The results demonstrated that the AUROC of RPE-R (0.771 ± 0.08) was significantly larger than the BMO-R (0.719 ± 0.009) for PPAA (+) results (Fig. 6 & Table 1).

RPEM also exhibited a significant relationship with BMOM (r = 0.607, p = 0.001 < 0.05). There was a significant difference in RPEM between eyes with “U”-shaped and “W”-shaped neural canals (Z = 2.004, p = 0.045 < 0.05). The difference in BMOM between eyes with the “U”-shaped and “W”-shaped neural canals was not statistically significant (Z = 1.244, p = 0.214 > 0.05).

## Discussion

VF testing is an important traditional method for the diagnosis of glaucoma, but the recognition of RNFLD in preperimetric glaucoma or the preglaucomatous stage has attracted increasing attention since the finding that perimetrically normal hemifields of glaucomatous eyes exhibit significantly lower macular GCC and cpRNFL thicknesses than the corresponding retinal regions of healthy eyes^19^. However, cpRNFL may not be a reliable indicator because the thickness of cpRNFL is normal in some eyes with preperimetric glaucoma^10^. The present study assumed that the subjects with typical wedge-shaped defects in red-free reflection imaging and the presence of absolute black cells in PPAA had glaucoma. Therefore, we investigated the characteristics of PPAA in eyes with preperimetric glaucoma and normal cpRNFL thickness. These eyes exhibited the typical wedge-shaped defects in red-free reflection and absolute black cells in PPAA, which are indicators of preperimetric glaucoma^7^. We also analysed the ONH morphology using EDI-SDOCT and introduced a new parameter, the RPE-R, as a complementary method to BMO measurements for the diagnosis of early glaucoma.

The measurement of normal retinal thickness was first described in the 1990s^20^, and its loss is associated with glaucoma^21^. The idea was primarily based on the progressive loss of RGCs during glaucomatous damage^22^. The symmetric distribution of retinal nerve fibres in reference to the fovea-disc axis is perturbed as glaucoma develops. Therefore, the glaucoma hemifield test in VF becomes a sensitive indicator of early glaucoma^23^. Asymmetric retinal thickness is a potentially valuable structural testing target for the diagnosis of glaucoma. Bagga *et al.*^24^ demonstrated that macular thickness asymmetry exhibited an AUROC of 0.76 to 0.84, with the potential to detect glaucomatous damage. The present study found that the distribution of absolute black cells in eight zones primarily centred on inferior Zones 4, 5, and 6 and superior Zones 5, 6, and 7, which coincided with RGC distribution. One explanation may be that the nasal to the macula areas convey fibres from the retina, both nasally and temporally, to the macula, whereas the temporal retina contains only the temporal fibres, resulting in a worse performance than the nasal area. The superior and inferior arcuate nerve fibres are also vulnerable to early glaucomatous changes^7^. Macular inner retinal layer thickness exhibits a significantly larger AUROC than cpRNFL thickness^25^. The subjects in the present study had a normal cpRNFL thickness, suggesting that injury of the RNFL in preperimetric glaucoma may occur at an earlier time in the macula than the circumpapillary area, which may be less sensitive^7^.

Chauhan *et al.*^26^ recently concluded that the clinically visible disc margin is an unreliable outer border of rim tissue compared to SD-OCT-based approaches for rim assessments because of the clinically and photographically invisible extensions of the BM. In contrast, BMO minimum rim width quantifies the neuroretinal rim from the true anatomical outer border and accounts for its variable trajectory at the point of measurement, and this measurement is also attributed to the introduction of the SD-OCT approach^27^. The present study further applied EDI-SDOCT to measure the minimal and horizontal distances between the BMO and ONH surfaces (i.e., BMOM and BMOH) and found that the ratio of BMOM to BMOH, termed as the BMO-R, in PPAA (+) eyes was significantly smaller than PPAA (−) eyes, which corresponded to optic nerve atrophy in our examinations. Measurements of the BMO cross-sectional diameter and the mean distance from the BMO plane to the anterior surface of the collagenous LC on the SD-OCT profile were strongly associated with matched histological measurements^28^. Therefore, BMO has become more popular for the assessment of ONH related-diseases^29^,^30^,^31^. We performed more quantitative assessments of these structures than previous studies. Notably, our study is the first study to measure and assess the RPE-R (i.e., the ratio of RPEM to RPEH). We further found that the AUROC of RPE-R (0.771 ± 0.08) was significantly larger than the BMO-R (0.719 ± 0.009) for PPAA (+) results, suggesting that the RPE-R may be a more sensitive and reliable parameter than traditional BMO-R for PPAA (+) and RNFLD detection. Therefore, RPE-R may serve as a reliable marker for the early diagnosis of glaucoma.

We also found that the morphology of the neural canal correlated with the PPAA results. Notably, eyes with “U”-shaped neural canals contained more absolute black cells. The classic posterior LC bowing and compression and excavation of the scleral canal wall beneath the BMO were observed in moderately or severely damaged glaucomatous eyes^32^. Downs *et al.*^15^ recently reported that profound deformations of the neural canal and the anterior-most aspect of the subarachnoid space architecture are observed at the onset of ONH surface changes in early experimental glaucomatous eyes of young adult monkeys. Nicholas *et al.*^33^ also found that the neuroretinal rim decreased and anterior LC surface depth increased significantly, but no change in RNFL thickness was detected. Similarly and additionally, we demonstrated these changes in human eyes with PPAA (+).

This study also has some limitations because of its retrospective design. We only analysed the absolute black cells in PPAA instead of calculating the varying levels of absolute black cells, and also only assessed the morphology of ONH in the vertical OCT profile. In addition, we did not analyse the correlation of ovality index (the tilt and shape of the optic nerve)^34^ with PPAA results and LC shape. All these will be improved in further researches.

In conclusion, we found that the distribution of absolute black cells in PPAA coincided with RGC distribution. There was a significant positive relationship between PPAA results and the morphology of the neural canal (“U” or “W” shaped). Eyes with a “U”-shaped neural canal contained more absolute black cells. Our study is the first study to measure and assess RPE-R, refering to the ratio of RPEM to RPEH. RPE-R may be a more sensitive and reliable parameter than traditional BMO-R for PPAA (+) and RNFLD detection. Therefore, RPE-R may serve as a reliable marker for the early diagnosis of glaucoma. Therefore, a “U”-shaped neural canal and lower BMO-R and RPE-R are proposed as novel early indicators of RNFLD in these glaucoma cases.

## Methods

### Patients

This retrospective study included patients who attended the Department of Ophthalmology - glaucomatous outpatient clinic at China Medical University. All patients had undergone stereoscopic disc examination, IOP measurement, refractive status (SE was calculated as the sum of the spherical plus half of the cylindrical error), A scan ultrasonography (axial length; AVISO, Quantel medical, France), visual field testing (central 30 degrees; Zeiss 750i Humphrey VF automated perimetry, Germany), and red-free reflection imaging (Spectralis HRA + OCT; Heidelberg Engineering, Heidelberg, Germany). Subjects also underwent SD-OCT (Spectralis HRA + OCT; Heidelberg Engineering, Heidelberg, Germany) examination to obtain cpRNFL thickness, PPAA and central vertical sectional imaging of ONH with EDI function. The raw images of each line scan in PPAA have been reviewed for the absence of artifacts. Two experienced and qualified reviewers (R.H. and X.L.M) separately assessed all red-free images to identify the RNFL losses according to the study of Seo *et al.*^10^. Discrepancies were referred to fundus specialists (K.Y. and R.G.) for final determination. At the same time, the ovality index defined as the ratio of the longest diameter to the shortest diameter of the optic disc was also measured in red-free images according to the study of Lee *et al.*^34^.

Subjects with normal cpRNFL thickness results defined as the global normal average measurement of the cpRNFL for each section (including temporal-superior (45°–90°), nasal-superior (90°–135°), nasal (135°–225°), nasal-inferior (225°–270°), temporal-inferior (270°–315°), and temporal (315°–45°)), typical wedge-shaped defects in red-free reflection imaging and any absolute black cells in PPAA were included in our study, and they did not receive any IOP-lowering treatment. Subjects were excluded if they had typical glaucomatous VF changes, IOP > 21 mmHg and a glaucomatous optic disc that exhibited increased cupping (vertical cup–disc ratio>0.6), vertical cup–disc ratio asymmetry of >0.2 between the two eyes, history of high myopia or other diseases affecting retinal thickness and ONH assessment, such as ischemic optic neuropathy, age-related macular degeneration, epimacular membrane or macular oedema.

The study adhered to the tenets of the Declaration of Helsinki, and the Medical Research Ethics Committee of China Medical University approved the study. Informed consent was obtained from all subjects.

### PPAA measurement

PPAA was performed in the macular 20° area of each eye using a 30° × 25° OCT volume scan with a colour scale representation of topographic retinal thickness. The posterior pole map provided information on the retinal thickness value of 64 (8 × 8) cells within each cell. The angle between the fovea and ONH centre relative to the horizontal axis was measured by FoDi correction line function of the Heidelberg Eye explorer software (version 1.5.12.0, Heidelberg Engineering, Heidelberg, Germany) automatically, simultaneously with cpRNFL detecting, and the negative angle was defined as the fovea was located below the level of the centre of the ONH, according to the study of Chauhan *et al.*^26^. The 8 × 8 grid was positioned symmetrically to the fovea-disc axis with the central point of the grid on the fovea, which was used for the inter-hemisphere comparisons.^7^ PPAA also provided a corresponding cell-to-cell comparison between hemispheres within the central 20°, and the differences are presented using a grey scale. Absolute black cells in PPAA refer to a retinal thickness difference of more than or equal to 30 μm, and this difference was recorded because it is difficult to clearly discern the values using a grey scale other than absolute black. Seo *et al.*^10^ referred to eyes with 2, 3, or 4 consecutive absolute black cells in PPAA as PPAA (+) and eyes with sporadic absolute black cells in PPAA as PPAA (−). Eight zones in the macular thickness map were defined. Each zone included reciprocal areas in the superior and inferior hemifield. We defined these zones according to Um *et al.*^7^, and three more zones were added in each hemifield.

### ONH measurement

ONH imaging was acquired using EDI-SDOCT. We defined the morphology of the neural canal including the anterior lamina cribrosa surface^16^,^33^,^35^ and excavation of the peripapillary sclera as “W” or “U” shaped according to the studies of Lee *et al.*^36^ and Rebolleda *et al.*^37^. Several experimental studies of enucleated human eyes also reported that posterior displacement was greatest in the middle of the lamina when an elevated IOP was applied, and the lamina assumed a U shape^38^. The minimal and horizontal distances between the BMO and ONH surfaces (BMOM and BMOH, respectively)^27^ were measured in the superior and inferior parts, respectively, using Heidelberg Eye explorer software (version 1.5.12.0, Heidelberg Engineering, Heidelberg, Germany) as the study of Chauhan *et al.*^26^. The terminal of RPE was used in Heidelberg Retina Tomograph III and Optovue RTVue OCT^38^ as part of the NCO, and we measured the minimal and horizontal distances between the terminal of RPE and ONH surfaces (RPEM and RPEH, respectively) similarly. The ratios of BMOM and BMOH (BMO-R) and RPEM and RPEH (RPE-R) were calculated.

A predictable pattern of axonal loss underlies early glaucomatous VF loss, which is ascribed to the inferior and superior poles of the ONH. Therefore, we chose the central cross-sectional image of ONH on OCT profile as a reference^39^,^40^.

### Statistical analysis

All analyses were performed using SPSS version 19.0 (Inc., Chicago, IL). The data were expressed as medians (Min-Max). Spearman’s rank correlation test was performed on the relationship between RPE-R and BMO-R and the relationship between the absolute black cells in PPAA and neural canal morphology. The area under the receiver operating characteristic curve (AUROC) was calculated to detect the diagnostic ability of RPE-R and BMO-R for PPAA. A probability (p) value of less than 0.05 was considered statistically significant.

## Additional Information

**How to cite this article**: Hua, R. *et al.* Detection of preperimetric glaucoma using Bruch membrane opening, neural canal and posterior pole asymmetry analysis of optical coherence tomography. *Sci. Rep.* **6**, 21743; doi: 10.1038/srep21743 (2016).

## Figures and Tables

**Table 1:** Measurement data of ONH.

Outline of neural canal	Value	
	“W” shape	52 eyes
	“U” shape	60 eyes
RPEH	Supra	528 (311.0–814.0) μm
	Infra	645 (369.0–1246.0) μm
RPEM	Supra	474.0(316.0–691.0) μm
	Infra	596.5 (361.0–863.0) μm
RPE-R		0.95 (0.74–1.02)
BMOH	Supra	396.0 (103.0–750.0) μm
	Infra	419.0 (298.0–1009.0) μm
BMOM	Supra	373.0 (82.0–605.0) μm
	Infra	411.0 (276.0–777.0) μm
BMO-R		0.94 (0.75–1.00)

ONH: optic nerve head; BMO: Bruch membrane opening; BMOH: the horizontal distances between the BMO and ONH surface; BMOM: the minimal distances between the BMO and ONH surface; BMO-R: the ratio of BMOM and BMOH; RPEH: the horizontal distances between the terminal of RPE and ONH surface; RPEM: the minimal distances between the terminal of RPE and ONH surface; RPE-R: the ratio of RPEM and RPEH.
